# Supplementary material for: Effects of Toxic Lithium Levels on ECG—Findings from the LiSIE Retrospective Cohort Study
Source: J Clin Med. 2022 Oct 8;11(19):5941. doi: 10.3390/jcm11195941 (PMC9572509; doi:10.3390/jcm11195941)
Supplement: Supplementary file 1 [file jcm-11-05941-s001.zip › jcm-1899312-supplementary.pdf]

File S1: STROBE Statement - checklist for our study

**STROBE requirement**	**#**	**Our study**
Title and abstract	1	
(a) Indicate the study's design with a commonly used term in the title and abstract		(a) Given: "Effects of toxic lithium levels on ECG – findings from the LiSIE retrospective cohort study"
(b) Provide in the abstract an informative and balanced summary of what was done and what was found		(b) Structured abstract provided.
Introduction		
Background/rationale: Explain the scientific background and rationale for the investigations being reported	2	Background outlined in introduction.
Objectives:<br>State specific objectives, including any prespecified hypotheses	3	Aims clearly stated in text, "We conducted the current study to examine the impact of toxic s-Li on the heart. Specifically, we tested the hypothesis that higher s-Li would lead to clinically relevant electrocardiogram (ECG) changes"
Methods		
Study design:<br>Present key elements of the study design early in the paper	4	Study design: Retrospective cohort study.<br>Key elements of the study included in the manuscript: study design, ethics and consent, participants, selection: inclusion and exclusion criteria, outcome definition, variable definitions, validation process, medical chart review, control for bias, missing data and statistical analysis.

Setting:<br>Describe the setting, locations, and relevant dates, including periods of recruitment, exposure, follow-up, and data collection	5	Setting and all relevant dates described in manuscript: "LiSIE invited all individuals in the Swedish regions of Västerbotten and Norrbotten of at least 18 years of age, who had either received a diagnosis of BD (ICD F31), schizoaffective disorder (SZD) (ICD F25), or who had used lithium as a mood stabiliser between 1997 and 2011. For the current study we included all patients from the LiSIE cohort who (a) lived in the Swedish region of Norrbotten, (b) had experienced a documented episode of lithium intoxication with s-Li ≥1.5 mmol/L at any time between 1997 and 2017, and (c) had an ECG recorded at the time of the intoxication. We excluded episodes of supratherapeutic s-Li when it was clear that these were only transient and had not given rise to an intoxication. This could occur for instance, when patients by mistake had taken their prescribed lithium before their blood- test. Lithium concentrations were obtained from a central laboratory database. ECGs were extracted from the electronic case records. Where necessary, we completed with data manually
		extracted from hardcopy case records. Prior to analysis, we anonymised the data"
Participants:<br>*(a)* Give the eligibility criteria, and the sources and methods of case ascertainment and control selection. Give the rationale for the choice of cases and controls<br>*(b)* For matched studies, give matching criteria and the number of controls per case	6	(a) As above<br>The medical records of all eligible patients were retrospectively reviewed for the outcomes and variables under study, from 1 January 1997 up to 31 December 2017**.**<br><br>(b) N/A.
Variables:<br>Clearly define all outcomes, exposures, predictors, potential confounders, and effect modifiers. Give diagnostic criteria, if applicable	7	Definition for exposures and variables given in text. The outcome of this study was ECG changes observed in the context of a lithium intoxication. Outcomes stratified by age and sex. Exposure variable was serum lithium concentrations, type of intoxication, concomitant use of medicines with potential QT prolonging effect, concomitant use of medicines with other cardiac effects, cardiovascular comorbidities and risk factors and potassium concentrations

Data sources /measurement:<br>For each variable of interest, give sources of data and details of methods of assessment (measurement). Describe comparability of assessment methods if there is more than one group	8	Definition for each variable given in text. Lithium concentrations were obtained from a central laboratory database. ECGs were extracted from the electronic case records. Where necessary, we completed with data manually extracted from hardcopy case records. Prior to analysis, we anonymised the data.
Bias:<br>Describe any efforts to address potential sources of bias	9	Potential sources of bias discussed, including selection and observer bias. We checked for selection bias in the LiSIE cohort. In accordance with the ethics approval granted, we compared age, sex, maximum recorded lithium, and creatinine concentrations in anonymous form for consenting and nonconsenting patients. There were no significant differences. For patients with lithium intoxication, we compared age and sex distribution between episodes with ECG and without ECG. Again, we did not find any significant differences. However, the mean lithium concentration was significantly higher in the group having an ECG available (p = 0.003). This means that our sample was biased to more severe lithium intoxications”
Study Size:	10	Cf. figure 1

Explain how the study size was arrived at		“Of 1136 patients exposed to lithium between 1997 and 2017, 92 patients had experienced 112 episodes of lithium intoxication with lithium concentrations ≥1.5 mmol/L. Seventeen patients had more than one episode of intoxication. An ECG at time of intoxication was available for 55 episodes in 50 patients. For 48 episodes, there was a reference ECG available. (Figure 1)”
Quantitative variables:<br>Explain how quantitative variables were handled in the analyses. If applicable, describe which groupings were chosen and why	11	The outcome of this study was ECG changes observed in the context of a lithium intoxication. During the 21-year review period, some patients had repeated episodes of lithium intoxication, which were not temporally related. Therefore, we analysed ECG changes per episode and not per patient. ECG selection described in detail in the text. Exposure variables described in detail in the text. Handling of variables described in statistical methods as below.

Statistical methods:<br>*a)* Describe all statistical methods, including those used to control for confounding *(b)* Describe any methods used to examine subgroups and interactions<br>(c) Explain how missing data were addressed<br>(d) If applicable, explain how matching of cases and controls was addressed<br>*(e)* Describe any sensitivity analyses	12	(a) We reported data at episode level. We conducted a descriptive analysis of all variables. For continuous variables, we reported mean and standard deviation (SD), as well as median and minimum/ maximum (min/max). For categorical variables, we reported proportions. The significance level was set to 0.05 throughout. The statistical analysis was conducted with IBM SPSS Statistics.<br>(b) For comparison of continuous variables between ECG at intoxication and subgroups, we used Wilcoxon rank test for paired data. For correlation between s-Li and HR or QTc at intoxication, we used Spearman Rank correlation to reflect the non-normal distribution of the data. For comparison of categorical variables between ECG at time of intoxication (ECGINTOX) and ECGPRE-INTOX/ECGPOST-INTOX, we used McNemar's test. We also conducted a univariate analysis with logistic regression to explore which exposure variables were associated with QT prolongation at intoxication.<br>(c) Addressed in the text. By default, ECG at time of intoxication was available for all included cases. Reference ECG were not available for all cases. We conducted separate subgroup-analyses for episodes with ECGINTOX and ECGPRE-INTOX or ECGPOST-INTOX. (d) N/A<br>(e) N/A
Results		
Participants:<br>*(a)* Report numbers of individuals at each stage of study—eg numbers potentially eligible, examined for eligibility, confirmed eligible, included in the study, completing follow-up, and analyzed<br>*(b)* Give reasons for nonparticipation at each stage<br>*(c)* Consider use of a flow diagram	13	(a+b) Of 1136 patients exposed to lithium between 1997 and 2017, 92 patients had experienced 112 episodes of lithium intoxication with lithium concentrations ≥1.5 mmol/L. Seventeen patients had more than one episode of intoxication. ECGINTOX was available for 55 episodes in 50 patients. For 48 episodes, there was a reference ECG available, 40 ECGPREINTOX and 28 ECGPOST-INTOX.<br><br>(c) Flow chart included in the manuscript as figure 1.

Descriptive data: *(a)* Give characteristics of study participants (e.g. demographic, clinical, social) and information on exposures and potential confounders *(b)* Indicate number of participants with missing data for each variable of interest	14	(a) Baseline characteristics described in table 3 of the manuscript.<br><br>(b) Included in the flow chart (figure 1) and in the text.
Outcome data: Report numbers in each exposure category, or summary measures of exposure	15	Outcome data presented in text, in tables 4-5 and figures 2-3.
Main results *(a)* Give unadjusted estimates and, if applicable, confounderadjusted estimates and their precision (eg, 95% confidence interval). Make clear which confounders were adjusted for and why they were included *(b)* Report category boundaries when continuous variables were categorized *(c)* If relevant, consider translating estimates of relative risk into absolute risk for a meaningful time period	16	(a) Results presented according to the statistical method outlined in item 12<br><br>(b) Results presented according to the statistical method outlined in item 12. Variable definitions given in method.<br><br>(c) N/A
Other analysis: Report other analyses done—e.g. analyses of	17	Subgroup-analysis between ECG at time of intoxication and available reference ECG (ECG PRE-INTOX, ECG POST-INTOX). Cf. 12. Univariate analysis for factors associated with QT prolongation at intoxication, presented in table 6.
subgroups and interactions, and sensitivity analyses		
Discussion		

Key results:<br>Summarize key results with reference to study objectives	18	Done
Limitations:<br>Discuss limitations of the study, taking into account sources of potential bias or imprecision. Discuss both direction and magnitude of any potential bias	19	Discussed in text. "Being observational, our current study depended on the quality of the information recorded in the medical records. ECG was available in only about half of all episodes, not all of which had reference ECG available. The quality was generally good. We only selected episodes with s-Li ≥1.5 mmol/L to avoid a bias towards mild and borderline intoxications. This bias towards more severe intoxications ensured that we did not underestimate the impact of lithium on cardiac conduction. The clinical course of lithium intoxications is often difficult at time of presentation. Early dialysis may prevent the development of more serious cardiac complications associated with higher lithium concentrations. In our region, the threshold for dialysis was low [7]. In our sample, dialysis was used as a treatment for intoxication in 11 episodes. The lowest s-Li at which dialysis was used was 1.9 mmol/L. Therefore, our findings should not be extrapolated to clinical settings that employ higher thresholds of s-Li for dialysis [22]."
Interpretation:<br>Give a cautious overall interpretation of results considering objectives, limitations, multiplicity of analyses, results from similar studies, and other relevant evidence	20	Results discussed in view of the limitations (weaknesses) of our study design and use of medical case records.
Generalisability: Discuss the generalizability (external validity) of the study results	21	Discussed in the context of bias, cf item 9.
Funding:<br>Give the source of funding and the role of the funders for the present study and, if applicable, for the original study on which the present article is based	22	This work was funded by grants (NLL-931604, NLL-941888, NLL-969413) from the Research & Development Fund of Norrbotten Region, Research and Innovation Unit, and the Department of Psychiatry, Sunderby Hospital, both Region Norrbotten, Sweden.<br><br>Conflict to interest statement for all authors included in manuscript.

Source: http://www.strobe-statement.org/. Accessed 16 May 2022
